# DNA replication origins in archaea

**DOI:** 10.3389/fmicb.2014.00179

**Published:** 2014-04-29

**Authors:** Zhenfang Wu, Jingfang Liu, Haibo Yang, Hua Xiang

**Affiliations:** ^1^State Key Laboratory of Microbial Resources, Institute of Microbiology, Chinese Academy of SciencesBeijing, China; ^2^University of Chinese Academy of SciencesBeijing, China

**Keywords:** DNA replication origin, origin recognition box, archaea, control, evolution, haloarchaea

## Abstract

DNA replication initiation, which starts at specific chromosomal site (known as replication origins), is the key regulatory stage of chromosome replication. Archaea, the third domain of life, use a single or multiple origin(s) to initiate replication of their circular chromosomes. The basic structure of replication origins is conserved among archaea, typically including an AT-rich unwinding region flanked by several conserved repeats (origin recognition box, ORB) that are located adjacent to a replication initiator gene. Both the ORB sequence and the adjacent initiator gene are considerably diverse among different replication origins, while *in silico* and genetic analyses have indicated the specificity between the initiator genes and their cognate origins. These replicator–initiator pairings are reminiscent of the *oriC-dnaA* system in bacteria, and a model for the negative regulation of origin activity by a downstream cluster of ORB elements has been recently proposed in haloarchaea. Moreover, comparative genomic analyses have revealed that the mosaics of replicator-initiator pairings in archaeal chromosomes originated from the integration of extrachromosomal elements. This review summarizes the research progress in understanding of archaeal replication origins with particular focus on the utilization, control and evolution of multiple replication origins in haloarchaea.

## INTRODUCTION

DNA replication is a fundamental cellular process that is functionally conserved across all three domains of life (bacteria, archaea, and eukaryote). The precise regulation of DNA replication ensures the accurate duplication of genomic information, and replication initiation is the first and most important stage of this regulation. The first model of DNA replication initiation was proposed for *Escherichia coli* in 1963, postulating that a trans-acting factor binds to a cis-acting site which triggers replication initiation (Jacob et al., 1963). In the subsequent 50 years, this “replicon model” has been demonstrated to be essentially true in all organisms, and the cis-acting site is now known as the replication origin. Bacterial chromosomes are typically replicated from a single origin, whereas the replication of eukaryotic chromosomes initiates from a number of discrete origins (Leonard and Mechali, 2013). DNA replication origins have been well-defined in bacteria and unicellular eukaryotes, and relative topics are covered in a number of excellent reviews (Messer, 2002; Mott and Berger, 2007; Zakrzewska-Czerwinska et al., 2007; Mechali, 2010; Aparicio, 2013). In contrast, focus on DNA replication origins in archaea, the third domain of life, commenced only approximately a decade ago. DNA replication origins have been mapped primarily for a few representatives of archaeal species distributed in the three main phyla, Euryarchaeota, Crenarchaeota, and Thaumarchaeota (Myllykallio et al., 2000; Lundgren et al., 2004; Robinson et al., 2004; Grainge et al., 2006; Norais et al., 2007; Majernik and Chong, 2008; Coker et al., 2009; Pelve et al., 2012, 2013; Wu et al., 2012, 2014). In addition, more detailed characterization has been revealed in several model systems, such as *Pyrococcus* species (Myllykallio et al., 2000; Matsunaga et al., 2001, 2003), *Sulfolobus* species (Lundgren et al., 2004; Robinson et al., 2004; Duggin et al., 2008; Samson et al., 2013), *Haloferax volcanii* (Norais et al., 2007; Hawkins et al., 2013) and *Haloarcula hispanica* (Wu et al., 2012, 2014). It is now known that archaea use a single or multiple origin(s) to replicate their circular chromosomes (Kelman and Kelman, 2004; Robinson and Bell, 2005; Hyrien et al., 2013). The multiple origins together with their adjacent initiator genes in certain archaeal chromosomes may have arisen from the capture of extrachromosomal elements and appear to be mosaics of distinct replicator–initiator pairings (Robinson and Bell, 2007; Wu et al., 2012).

This replicator–initiator system consists of an origin region and an initiator gene (the *cdc6* gene in most cases and *whiP* in the *oriC3* of *Sulfolobus* species). The origin region usually has a high content of adenine and thymine residues (AT-rich) flanked by several conserved repeated motifs known as origin recognition boxes (ORBs). The initiator protein Cdc6 (also denoted Orc or Orc1/Cdc6 in other papers) shows homology to both Orc1 and Cdc6 of eukaryotes and therefore is considered to be involved in both specific recognition of the origin region and loading of the minichromosome maintenance helicase (MCM; Robinson and Bell, 2005). Despite the conservation of the replicator-initiator structure, archaeal replication origins exhibit considerable diversity in terms of both the ORB elements within different origins and their adjacent initiator genes. The specificity of the initiator genes and their cognate origins was recently established by means of *in silico* and genetic analyses in *Sulfolobus* species (Samson et al., 2013) and *Haloarcula hispanica* (Wu et al., 2012, 2014). The *cis* organization of the replication origin and the initiator gene (replicator–initiator) is reminiscent of the *oriC-dnaA* system in bacteria. Recently, we revealed that bacterial-like control mechanisms may be used by different replication origins in haloarchaea, and a model has been proposed for the negative regulation of *oriC2* by a downstream cluster of ORB elements in *Haloarcula hispanica* (Wu et al., 2014).

The goal of this review is to present an overview of progress made over the past decade in our understanding of DNA replication origins of archaeal genomes, including the identification (mapping), characterization and evolution of multiple replication origins on the chromosomes. We focus on the utilization and control of multiple replication origins in haloarchaea, as well as comparisons of replication origins from different archaeal species to draw the generality and evolution of multiple replication origins in archaea.

## IDENTIFICATION (MAPPING) OF REPLICATION ORIGINS

Similar to bacteria, archaea have simple circular chromosomes (and also several extrachromosomal elements in some archaea); however, many archaea characterized to date harbor multiple replication origins. The approaches developed in bacteria or eukaryotes have been employed to investigate replication origins in archaea, such as GC-skew analysis (Myllykallio et al., 2000), the Z-curve method (Zhang and Zhang, 2003), autonomously replicating sequence (ARS) assay (Berquist and DasSarma, 2003; Norais et al., 2007; Wu et al., 2012), replication initiation point mapping (RIP mapping; Matsunaga et al., 2003), two-dimensional gel analysis (Matsunaga et al., 2001; Robinson et al., 2004), and marker frequency analysis (MFA; Lundgren et al., 2004; Coker et al., 2009; Pelve et al., 2012; Hawkins et al., 2013; Wu et al., 2014). DNA replication origins have been mapped in about a dozen archaeal species.

### SINGLE REPLICATION ORIGIN IN *Pyrococcus* SPECIES

**FIGURE 1:**
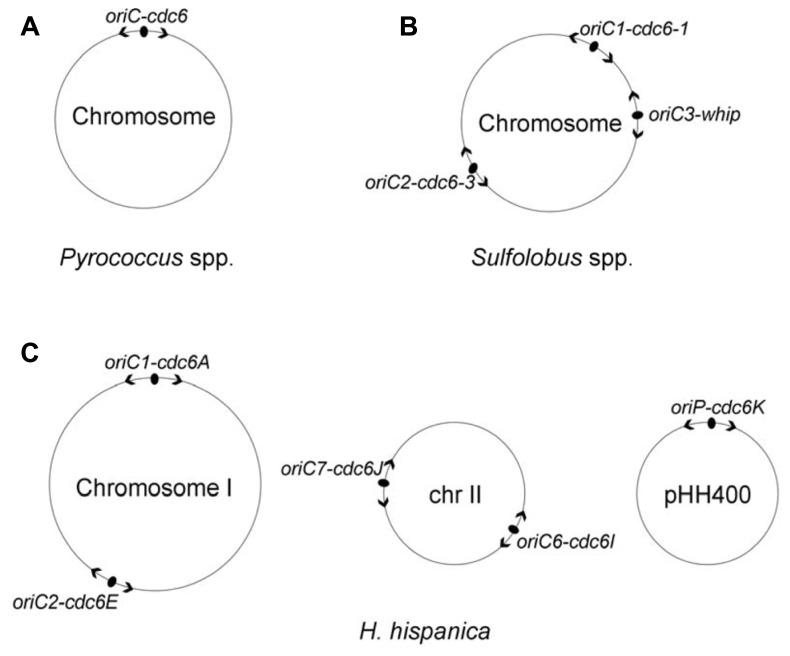
**Distribution of DNA replication origins in three well-studied archaeal model systems, *Pyrococcus* species **(A)**, *Sulfolobus* species **(B)** and *Haloarcula hispanica***(C)**.** Origins are indicated with filled ovals, and arrowheads designate bidirectional replication. Replicator-initiator indicates that each origin is specified by its proximally encoded initiator. Both *Pyrococcus* species and *Sulfolobus* species contain a single chromosome; the chromosome of *Pyrococcus* species is replicated from a single origin (Myllykallio et al., 2000), whereas the chromosome of *Sulfolobus* species is replicated from three origins in near synchrony (Duggin et al., 2008). The *Haloarcula hispanica* genome consists of a main chromosome and two extrachromosomal elements with five active replication origins: *oriC1*-*cdc6A* and* oriC2*-*cdc6E* in the main chromosome I, *oriC6*-*cdc6I* and *oriC7*-*cdc6J* in the minichromosome II, and *oriP*-*cdc6K* in the megaplasmid pHH400 (Wu et al., 2012).

The first description of DNA replication origins of archaeal genomes was reported by Myllykallio et al. (2000). These researchers identified a single replication origin (*oriC*) in *Pyrococcus abyssi* by means of cumulative skew of GGGT, and the study found that the *oriC* is flanked with the only *cdc6* gene and several eukaryotic-like replication genes (Myllykallio et al., 2000). The origin organization was observed to be highly conserved in two other *Pyrococcus* species, *Pyrococcus horikoshii* and *Pyrococcus furiosus* (Myllykallio et al., 2000). The *oriC* was then experimentally confirmed via two-dimensional (2D) gel analysis (Matsunaga et al., 2001) and RIP mapping (Matsunaga et al., 2003), and the studies demonstrated that the *oriC* has several repeated sequences (now named ORBs) and is directly upstream of the *cdc6* gene, reminiscent of the *oriC*-*dnaA* origin system in bacteria. Furthermore, the specific interaction of the Cdc6 protein with the *oriC* was detected via chromatin immunoprecipitation assays (Matsunaga et al., 2001). All of these data indicated that the circular chromosome of the *Pyrococcus* species is bidirectionally replicated from a bacterial mode of replication origin by eukaryotic-type machinery (**Figure 1A**).

### THREE REPLICATION ORIGINS IN *Sulfolobus* SPECIES

The first example of archaeal chromosomes with multiple replication origins was the identification of three replication origins in the single chromosome of *Sulfolobus* species using 2D gel analysis (Robinson et al., 2004, 2007) and microarray-based MFA (Lundgren et al., 2004), and the results demonstrated that bidirectional replication initiated from three origins in both *Sulfolobus acidocaldarius* and* Sulfolobus solfataricus* (*oriC1*, *oriC2*, and *oriC3*; **Figure 1B**). The *oriC1* and *oriC2*, in each species, are located directly upstream of *cdc6-1* and *cdc6-3*, respectively, which have previously been identified by 2D gel electrophoresis in *S. solfataricus* (Robinson et al., 2004). The third origin, *oriC3*, is adjacent to the *whiP* (Winged-helix initiator protein) gene that is related to the eukaryotic *cdt1* gene. An origin comparison between *Aeropyrum* and *Sulfolobus* suggested that the *oriC3*-*whiP* might have originated from the capture of extrachromosomal elements (Robinson and Bell, 2007). Using synchronized cultures, MFA results indicated that all three origins fire once per cell cycle and are initiated in near synchrony but with a slightly later activation of *oriC2* (Lundgren et al., 2004; Duggin et al., 2008). Recently, three replication origins were also mapped in another *Sulfolobus* species, *Sulfolobus islandicus*, and a combination of genetic and MF analyses demonstrated that the three origins are specifically dependent on their adjacent initiator genes (two *cdc6* genes and one *whiP* gene; Samson et al., 2013).

### MULTIPLE REPLICATION ORIGINS IN HALOARCHAEA

Haloarchaeal genomes are generally composed of multiple genetic elements (chromosome, minichromosome, and megaplasmids) with multiple Cdc6 homologs (Capes et al., 2011), which is suggestive of the occurrence of multiple replication origins. Recently, multiple replication origins were predicted in 15 completely sequenced haloarchaeal genomes by searching for putative ORBs associated with *cdc6* genes (Wu et al., 2012), and active replication origins have been experimentally studied in three model systems, *Halobacterium* sp. NRC-1 (Berquist and DasSarma, 2003; Coker et al., 2009), *Haloferax volcanii* (Norais et al., 2007; Hawkins et al., 2013) and *Haloarcula hispanica* (Wu et al., 2012, 2014).

The first prediction of multiple DNA replication origins in haloarchaeal genomes came from Z curve method analysis of the genome of *Halobacterium* sp. NRC-1, which revealed two *cdc6*-adjacent replication origins in its chromosome (Zhang and Zhang, 2003). However, only one replication origin was verified to have ARS activity (Berquist and DasSarma, 2003). Whole-genome MFA was employed to map the activation of replication origins *in vivo* in *Halobacterium* sp. NRC-1, which demonstrated multiple discrete origin sites in the chromosome, with two being located in the vicinity of *cdc6* genes (denoted *orc7* and *orc10* in the original paper; Coker et al., 2009).

Eleven *cdc6* genes are encoded in *Haloarcula hispanica*, and eight of them have surrounding ORB-like elements. ARS activity assays demonstrated that only five predicted origins, *oriC1*-*cdc6A* and *oriC2*-*cdc6E* in the main chromosome, *oriC6*-*cdc6I*, and *oriC7*-*cdc6J* in the minichromosome and *oriP-cdc6K* in the megaplasmid (pHH400), were able to confer replication ability to a non-replicating plasmid (**Figure 1C**; Wu et al., 2012). Recently, we combined extensive gene deletion and microarray-based MFA to map the activation of replication origins *in vivo* in *Haloarcula hispanica*, demonstrating that the chromosome is bidirectionally replicated from the two initially proven origins, *oriC1*-*cdc6A*, and *oriC2*-*cdc6E* (Wu et al., 2014). Importantly, our results indicated that one active *ori-cdc6* pairing on each replicon, i.e., *oriC1-cdc6A* or *oriC2-cdc6E* on the chromosome, *oriC6-cdc6I* or *oriC7-cdc6J* on the minichromosome, and *oriP-cdc6K* on pHH400, is essential for genome replication in *Haloarcula hispanica* (Wu et al., 2014).

Five replication origins were initially identified in *Haloferax volcanii* using a combination of bioinformatics and genetic approaches: two within the chromosome and one each within the three megaplasmids pHV1, pHV3, and pHV4 (Norais et al., 2007). Recently, aside from the previously identified origins, a sixth replication origin was mapped in the chromosome via high-throughput sequencing-based MFA (Hawkins et al., 2013). All six replication origins are adjacent to *cdc6* genes. Furthermore, four chromosomal replication origins were mapped in the laboratory H26 strain with integration of pHV4 into the chromosome (Hawkins et al., 2013). Surprisingly, the four origins can be deleted simultaneously, and in the absence of these replication origins, the strain even grew 7.5% faster than the wild-type strain (Hawkins et al., 2013). Because the *radA* gene (the archaeal *recA*/*rad51* homologue) was determined to be essential in the absence of all four origins, the authors proposed that the replication of the origin-less *Haloferax volcanii* chromosome is dependent on homologous recombination (Hawkins et al., 2013). However, this mode of recombination-dependent replication of the *Haloferax volcanii* chromosome was not yet observed in other investigated archaea. In contrast, at least one active replication origin has been proven to be essential for chromosome replication in *Haloarcula hispanica* (Wu et al., 2014), and triple-deletion mutant was not available for the three initiators in the chromosome of *S. islandicus* (Samson et al., 2013). It would be interesting to investigate how the RadA-dependent replication (if any) efficiently replicates the *Haloferax volcanii* chromosome, or if there are undetected replication origins functioned in the chromosome lacking the main origins.

### MAPPING OF REPLICATION ORIGINS IN OTHER ARCHAEA

DNA replication origins have been well-defined in several bacterial model systems, and have been predicted and/or identified in more than 1300 bacterial genomes (Gao and Zhang, 2007, 2008). Similarly, to understand the general nature of replication origins in archaea, it is necessary to determine and compare replication origins from a broad selection of archaeal species. Fortunately, the genomes of 100s of archaea distributed in different phyla have been sequenced and are publically available, allowing the prediction and mapping of replication origins in these genomes. To date, replication origins have been demonstrated in a dozen archaeal species. Similar to *Pyrococcus* species, *Archaeoglobus fulgidus* has been shown to contain a single replication origin (Maisnier-Patin et al., 2002). Two replication origins have been identified in *Aeropyrum pernix* by using a combination of biochemical and two-dimensional gel electrophoresis (Grainge et al., 2006; Robinson and Bell, 2007). Studies of DNA replication in methanogens have demonstrated that a single origin is responsible for replication initiation of the chromosome of *Methanothermobacter thermautotrophicus* (Capaldi and Berger, 2004; Majernik and Chong, 2008). Recently, four replication origins were mapped in the single chromosome of *Pyrobaculum calidifontis* via high-throughput sequencing-based MFA (Pelve et al., 2012). To generate a broader view of modes of origin replication in archaea, Pelve et al. (2013) further completed origin mapping in a thaumarchaeon, revealing a single replication origin in the *Nitrosopumilus maritimus* chromosome.

## DISTINCT REPLICATOR-INITIATOR SYSTEMS IN ARCHAEA

The initiator protein DnaA is highly conserved in bacteria, and bacterial replication origins generally possess conserved sequence elements, DnaA boxes. In contrast, the three replication origins in *Sulfolobus* species differ from each other. Each of the three origins is specifically recognized by its proximally encoded initiator protein, two distinct Cdc6 proteins and WhiP (Dueber et al., 2011; Samson et al., 2013). In addition, the recognition mechanisms appear to be different, as classic ORB and its shorter version (miniORB) are, respectively, observed in the *oriC1* and *oriC2* regions, while neither is observed in the *oriC3* region (Robinson et al., 2004; Samson et al., 2013).

Haloarchaeal genomes generally contain multiple *cdc6* genes and replication origins. Recently, we conducted a comparison of the origin-associated Cdc6 homologs and the corresponding predicted ORB elements. Our results suggested that the replication origins from haloarchaeon are notably diverse in terms of ORB elements and their adjacent *cdc6* genes, which could be sorted into distinct families. Based on this phylogenetic analysis, linkage-specificity of Cdc6 homologs and the corresponding ORB elements was proposed, suggestive of their specific interaction (Wu et al., 2012). Very recently, we employed comprehensive genetic studies to investigate the specificity of multiple replication origins and *cdc6* genes in *Haloarcula hispanica*, and our results indicated that each Cdc6 protein specifically recognizes its proximal origin (Wu et al., 2014). Thus, multiple replication origins along with their adjacent *cdc6* genes appear to be distinct *ori-cdc6* systems. These distinct *ori-cdc6* systems in haloarchaeon may have many evolutionary advantages: first, it ensures the compatibility of multiple replication origins, which accounts for the observations that multiple Cdc6 proteins from a haloarchaeal genome are distributed into different families (Wu et al., 2012) and that the *oriC2*-containing plasmid is incompatible with *Haloarcula hispanica* (Wu et al., 2014); second, distinct *ori-cdc6* pairings help minimize competition among multiple origins for initiators and maintain independent control of replication initiation at different origins. Importantly, as haloarchaeal genomes generally contain multiple replicons, distinct *ori-cdc6* origins may be favorable for replicon-specific replication control, similar to the different modes of replication origin adopted by the two chromosomes of *Vibrio cholerae* (Egan and Waldor, 2003).

To understand the molecular mechanisms involved in the specific recognition of origins by initiators, the structures of two origin-bound Cdc6 proteins from *Aeropyrum pernix* (Gaudier et al., 2007) and *S. solfataricus* (Dueber et al., 2007) were crystallized. Both of the two Cdc6 proteins contain an N-terminal AAA^+^ domain and a C-terminal WH domain. Intriguingly, both of the studies demonstrated that, in addition to the canonical DNA binding WH domain, the AAA^+^ domains of these two initiators are responsible for recognizing origins (Dueber et al., 2007; Gaudier et al., 2007). Subsequently, biochemical data also demonstrated that both the WH domain and AAA^+^ domain contribute to the origin-binding specificity of the Cdc6 protein (Dueber et al., 2011).

## CONTROL OF REPLICATION INITIATION AT MULTIPLE ORIGINS IN ARCHAEA

Multiple mechanisms that regulate replication initiation have been well-characterized in both bacteria and unicellular eukaryotes, and are summarized in a number of excellent reviews (Mott and Berger, 2007; Mechali, 2010; Rajewska et al., 2012; Aparicio, 2013). In contrast, the mechanisms by which archaea regulate replication initiation at multiple origins, either on the same chromosome or from different genetic elements, are far less understood. All of the archaeal replication origins characterized to date are dependent on their adjacent initiator gene (the *cdc6* gene in most cases; Samson et al., 2013; Wu et al., 2014), and these distinct *ori-cdc6* pairings may contribute to their independent control. In addition, the *cis* location of the *cdc6* gene and the origin is proved to not be required for ARS activity in both *Haloferax volcanii* and *Haloarcula hispanica* (Norais et al., 2007; Wu et al., 2014). Therefore, we have proposed that direct linkage of the initiator gene to the origin may facilitate its transcription after replication initiation to sequentially control its cognate origin.

Using the *Haloarcula hispanica* model system, we suggested that some bacterial-like mechanisms may be employed at different replication origins in haloarchaea (Wu et al., 2014). A G-rich inverted-repeat directly inside each ORB element of *Haloarcula hispanica oriC1* was shown to be a replication enhancer that stimulated origin activation at *oriC1*. Because of the repeat’s close location to ORB elements, we proposed that the G-rich inverted-repeat enhances the binding of initiator or regulatory factors at *oriC1*, similar to many repeated sequences in bacteria that are binding sites for initiation proteins or regulatory factors, playing a crucial role in the control of replication initiation (Rajewska et al., 2012). In addition, a model has been proposed, and partly tested, for the negative regulation of *oriC2* by a downstream cluster of Cdc6 binding elements (ORBs), likely *via* Cdc6E titration, similar to the negative control of replication initiation via a *datA* locus exhibiting DnaA-titrating activity in *E. coli* (Kitagawa et al., 1998). More interestingly, many additional predicted replication origins have the *oriC2*-like structure, suggesting that this strategy of negative replication origin control is used generally by haloarchaea.

Despite the bacterial-like structure of archaeal replication origins, archaea use eukaryotic-type replication machinery (Robinson and Bell, 2005), indicating that archaea may adopt eukaryotic-like mechanisms to control replication proteins and thus replication initiation. Interestingly, genome-wide transcription mapping indicated that serine–threonine protein kinases show cyclic induction in *Sulfolobus* species, indicating that regulatory factors similar to eukaryotic cyclin-dependent kinase (CDK) complexes may be present in archaea (Lundgren and Bernander, 2007). Recently, an ATP-ADP binary switch model for Cdc6-mediated replication control was proposed in *S. islandicus*, postulating that binding of ATP remodels Cdc6 conformation for efficient MCM recruitment, and subsequent ATP hydrolysis renders Cdc6 incapable of further recruiting MCM (Samson et al., 2013). In addition, as almost all replication origins are dependent on Cdc6 proteins, conformational changes of Cdc6 proteins may play important roles in coordinating replication initiation at different origins within a cell.

## EVOLUTION OF MULTIPLE REPLICATION ORIGINS IN ARCHAEA

**FIGURE 2:**
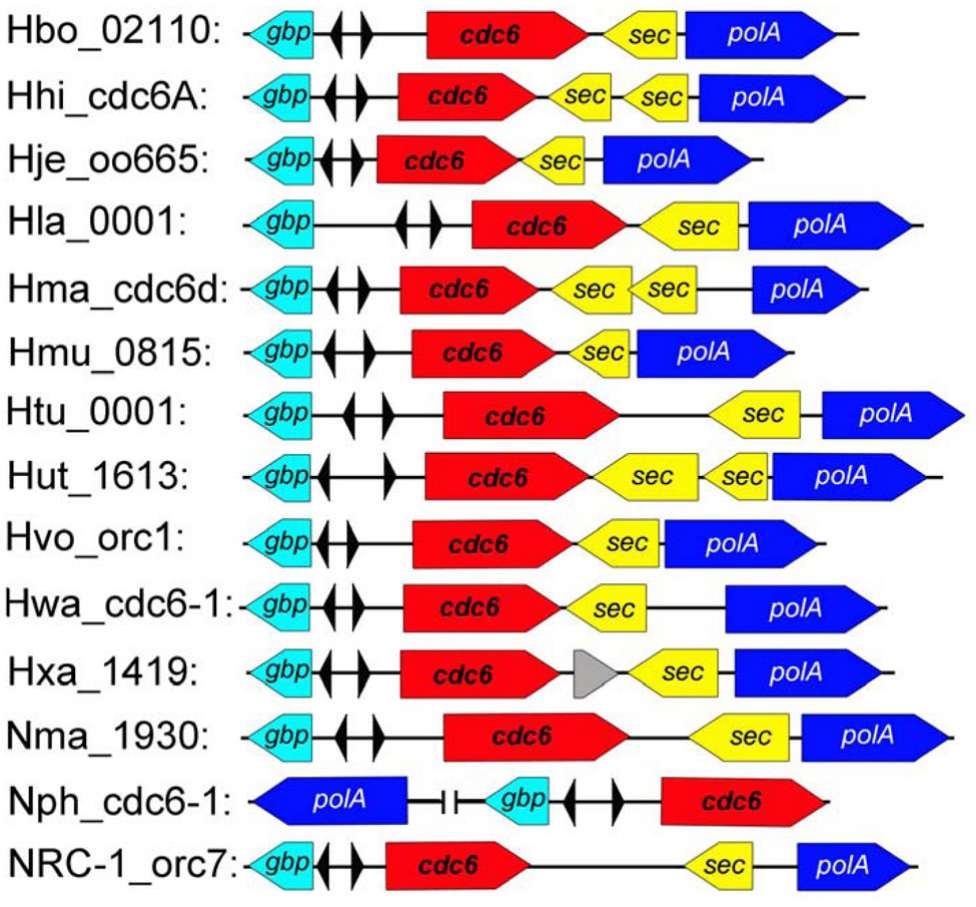
**The conserved *oriC1* origin of replication in sequenced haloarchaeal genomes.** The *oriC1* context region was mapped as shown in the sequenced haloarchaea. The colored boxed arrows represent different genes as follows: GTP-binding protein (*gbp*, teal), initiator protein (*cdc6*, red), signal sequence peptidase (*sec*, yellow) and DNA-directed DNA polymerase (*polA*, blue). The inverted ORB elements are indicated by small triangles.

Although considerable diversity of replication origins has been observed in haloarchaea, comparison analysis revealed a conserved replication origin, *oriC1*, which is positioned in the main chromosome of all analyzed haloarchaeal genomes (Coker et al., 2009; Wu et al., 2012). Both the ORBs within *oriC1* and the *oriC1*-associated Cdc6 homologs are highly conserved. In addition, gene order analysis found that genes around *oriC1* are highly syntenic among haloarchaea (**Figure 2**; Capes et al., 2011). Notably, other studies (Robinson et al., 2004; Coker et al., 2009) and our results indicated that the *oriC1* replication origin is broadly conserved in archaea, in terms of both function and structure, which strongly suggested that the ancestral chromosome was dependent on *oriC1*. Variations were observed in *oriC1* homologs from different archaeal phyla, which may contribute to the adaptability of archaea to different extreme environments. For example, an extended halophile-specific “G-string” element has been identified at the end of each ORB in haloarchaea, and these “G-string” elements have been proven to be essential for autonomous replication based on the *oriC1* in *Haloarcula hispanica* (Wu et al., 2014).

Multiple replication origins along with their adjacent *cdc6* genes appear to be mosaics of distinct replicator–initiator systems. A comparison between *Aeropyrum* and *Sulfolobus* origins suggested that the capture of extrachromosomal elements accounts for replicon evolution (Robinson and Bell, 2007). In particular, it has been proposed that the three replication origins of the *Sulfolobus* species arose by the integration of extrachromosomal elements into a single-origin ancestral chromosome (*oriC1-cdc6-1*), and the acquisition of *oriC3-whiP* occurred prior to the integration of *oriC2-cdc6-3* (Samson et al., 2013). Similarly, genomic context analyses of *ori-cdc6* systems in haloarchaea revealed that 40% of predicted replication origins were observed with transposases or integrases nearby, indicative of the translocation of a subset of replication origins among haloarchaea. In addition, comparative analyses of the selected replication origins suggested that different evolutionary mechanisms, including ancestral conservation and coupled acquisition and deletion events, may account for the current mosaics of multiple replication origins in the haloarchaeal genomes. Importantly, a comparative genomic analysis of two *Haloarcula* species, *Haloarcula hispanica* and *Haloarcula marismortui*, revealed that the species-specific origins are located in extremely variable regions, suggesting that these novel origins were recently acquired, via either integration into the chromosome or rearrangement of extrachromosomal elements (Wu et al., 2012). Further work may focus on comparisons of replication origins from closely related species to reveal the dynamics of origin evolution and whether origin evolution alters the mode of genomic replication.

## PERSPECTIVES

To date, the number of archaea with mapped replication origins is still limited, which to some extent has affected us to get a panoramic view of the generality and evolution of replication origins in archaea. In addition to the mapping of replication origins, the development of prediction algorithms for replication origins in archaeal genomes and the construction of databases with these predicted origins (Gao et al., 2013) will be useful for comparing replication origins from a broader range of archaeal species. Fortunately, the rapid increase in the number of complete archaeal genomic sequences that are publically available will promote our studies of archaeal replication origins.

In addition, the control and coordination of replication initiation at multiple origins in archaea is far less understood. The multireplicon structure of haloarchaeal genomes allows for precise control and coordination of replication initiation at multiple origins. As the chromosome and extrachromosomal elements within a haloarchaeon are generally different sizes and have different copy numbers (Breuert et al., 2006; Liu et al., 2013), it will be interesting to reveal whether they initiate synchronously and how they maintain different copy numbers, as well as what roles multiple replication origins play in governing polyploidy in haloarchaea. In addition, the coordination of multiple origins may play important roles in maintaining the multireplicon structure of haloarchaeal genomes. As most replication origins are dependent on Cdc6 proteins in haloarchaea (excluding the origins of small plasmids), we propose that the coordination of replication initiation at different origins may be obtained by conformational changes of Cdc6 proteins via an ATP-ADP binary switch, which has recently been proposed for chromosome replication in *S. islandicus* (Samson et al., 2013). Thus, more exhaustive work should be taken into account to uncover the control and coordination of the replication initiation from multiple origins, either on the same chromosome or from different genetic elements, in haloarchaeal multireplicon genomes.

## Conflict of Interest Statement

The authors declare that the research was conducted in the absence of any commercial or financial relationships that could be construed as a potential conflict of interest.

## References

[B1] Aparicio O. M. (2013). Location, location, location: it’s all in the timing for replication origins. *Genes Dev.* 27 117–128 10.1101/gad.209999.112 23348837 PMC3566304

[B2] Berquist B. R. DasSarma S. (2003). An archaeal chromosomal autonomously replicating sequence element from an extreme halophile, *Halobacterium* sp. strain NRC-1. *J. Bacteriol.* 185 5959–5966 10.1128/JB.185.20.5959-5966.2003 14526006 PMC225043

[B3] Breuert S. Allers T. Spohn G. Soppa J. (2006). Regulated polyploidy in halophilic archaea. *PLoS ONE* 1:e92. 10.1371/journal.pone.0000092 PMC1762399 17183724

[B4] Capaldi S. A. Berger J. M. (2004). Biochemical characterization of Cdc6/Orc1 binding to the replication origin of the euryarchaeon *Methanothermobacter thermoautotrophicus*. *Nucleic Acids Res.* 32 4821–4832 10.1093/nar/gkh819 15358831 PMC519113

[B5] Capes M. D. Coker J. A. Gessler R. Grinblat-Huse V. Dassarma S. L. Jacob C. G. (2011). The information transfer system of halophilic archaea. *Plasmid* 65 77–101 10.1016/j.plasmid.2010.11.005 21094181

[B6] Coker J. A. Dassarma P. Capes M. Wallace T. Mcgarrity K. Gessler R. (2009). Multiple replication origins of *Halobacterium* sp. strain NRC-1: properties of the conserved *orc7*-dependent *oriC1*. *J. Bacteriol.* 191 5253–5261 10.1128/JB.00210-09 19502403 PMC2725591

[B7] Dueber E. C. Costa A. Corn J. E. Bell S. D. Berger J. M. (2011). Molecular determinants of origin discrimination by Orc1 initiators in archaea. *Nucleic Acids Res.* 39 3621–3631 10.1093/nar/gkq1308 21227921 PMC3089459

[B8] Dueber E. L. Corn J. E. Bell S. D. Berger J. M. (2007). Replication origin recognition and deformation by a heterodimeric archaeal Orc1 complex. *Science* 317 1210–1213 10.1126/science.1143690 17761879

[B9] Duggin I. G. Mccallum S. A. Bell S. D. (2008). Chromosome replication dynamics in the archaeon *Sulfolobus acidocaldarius*. *Proc. Natl. Acad. Sci. U.S.A.* 105 16737–16742 10.1073/pnas.0806414105 18922777 PMC2575489

[B10] Egan E. S. Waldor M. K. (2003). Distinct replication requirements for the two *Vibrio cholerae* chromosomes. *Cell* 114 521–530 10.1016/S0092-8674(03)00611-1 12941279

[B11] Gao F. Luo H. Zhang C. T. (2013). DoriC 5.0: an updated database of *oriC* regions in both bacterial and archaeal genomes. *Nucleic Acids Res.* 41 D90–D93 10.1093/nar/gks990 23093601 PMC3531139

[B12] Gao F. Zhang C. T. (2007). DoriC: a database of *oriC* regions in bacterial genomes. *Bioinformatics* 23 1866–1867 10.1093/bioinformatics/btm255 17496319

[B13] Gao F. Zhang C. T. (2008). Ori-Finder: a web-based system for finding *oriC*s in unannotated bacterial genomes. *BMC Bioinformatics*9:79 10.1186/1471-2105-9-79 PMC2275245 18237442

[B14] Gaudier M. Schuwirth B. S. Westcott S. L. Wigley D. B. (2007). Structural basis of DNA replication origin recognition by an ORC protein. *Science* 317 1213–1216 10.1126/science.1143664 17761880

[B15] Grainge I. Gaudier M. Schuwirth B. S. Westcott S. L. Sandall J. Atanassova N. (2006). Biochemical analysis of a DNA replication origin in the archaeon *Aeropyrum pernix*. *J. Mol. Biol.* 363 355–369 10.1016/j.jmb.2006.07.076 16978641

[B16] Hawkins M. Malla S. Blythe M. J. Nieduszynski C. A. Allers T. (2013). Accelerated growth in the absence of DNA replication origins. *Nature* 503 544–547 10.1038/nature12650 24185008 PMC3843117

[B17] Hyrien O. Rappailles A. Guilbaud G. Baker A. Chen C. L. Goldar A. (2013). From simple bacterial and archaeal replicons to replication N/U-domains. *J. Mol. Biol.* 425 4673–4689 10.1016/j.jmb.2013.09.021 24095859

[B18] Jacob F. Cuzin F. Brenner S. (1963). On Regulation of DNA Replication in Bacteria. *Cold. Spring Harb. Symp. Quant. Biol.* 28 329–348 10.1101/SQB.1963.028.01.048

[B19] Kelman L. M. Kelman Z. (2004). Multiple origins of replication in archaea. *Trends Microbiol.* 12 399–401 10.1016/j.tim.2004.07.001 15337158

[B20] Kitagawa R. Ozaki T. Moriya S. Ogawa T. (1998). Negative control of replication initiation by a novel chromosomal locus exhibiting exceptional affinity for *Escherichia coli* DnaA protein. *Genes Dev.* 12 3032–3043 10.1101/gad.12.19.3032 9765205 PMC317192

[B21] Leonard A. C. Mechali M. (2013). DNA replication origins. *Cold Spring Harb. Perspect. Biol.* 5:a010116 10.1101/cshperspect.a010116 PMC3783049 23838439

[B22] Liu X. Miao D. Zhang F. Wu Z. Liu J. Xiang H. (2013). Characterization of the minimal replicon of pHM300 and independent copy number control of major and minor chromosomes of *Haloferax mediterranei*. *FEMS Microbiol. Lett.* 339 66–74 10.1111/1574-6968.12052 23173581

[B23] Lundgren M. Andersson A. Chen L. Nilsson P. Bernander R. (2004). Three replication origins in *Sulfolobus* species: synchronous initiation of chromosome replication and asynchronous termination. *Proc. Natl. Acad. Sci. U.S.A.* 101 7046–7051 10.1073/pnas.0400656101 15107501 PMC406463

[B24] Lundgren M. Bernander R. (2007). Genome-wide transcription map of an archaeal cell cycle. *Proc. Natl. Acad. Sci. U.S.A.* 104 2939–2944 10.1073/pnas.0611333104 17307872 PMC1815285

[B25] Maisnier-Patin S. Malandrin L. Birkeland N. K. Bernander R. (2002). Chromosome replication patterns in the hyperthermophilic euryarchaea *Archaeoglobus fulgidus* and *Methanocaldococcus* (*Methanococcus*) *jannaschii*. *Mol. Microbiol.* 45 1443–1450 10.1046/j.1365-2958.2002.03111.x 12207709

[B26] Majernik A. I. Chong J. P. (2008). A conserved mechanism for replication origin recognition and binding in archaea. *Biochem. J.* 409 511–518 10.1042/BJ20070213 17956224

[B27] Matsunaga F. Forterre P. Ishino Y. Myllykallio H. (2001). *In vivo* interactions of archaeal Cdc6/Orc1 and minichromosome maintenance proteins with the replication origin. *Proc. Natl. Acad. Sci. U.S.A.* 98 11152–11157 10.1073/pnas.191387498 11562464 PMC58699

[B28] Matsunaga F. Norais C. Forterre P. Myllykallio H. (2003). Identification of short “eukaryotic” Okazaki fragments synthesized from a prokaryotic replication origin. *EMBO Rep.* 4 154–158 10.1038/sj.embor.embor732 12612604 PMC1315830

[B29] Mechali M. (2010). Eukaryotic DNA replication origins: many choices for appropriate answers. *Nat. Rev. Mol. Cell Biol.* 11 728–738 10.1038/nrm2976 20861881

[B30] Messer W. (2002). The bacterial replication initiator DnaA. DnaA and *oriC*, the bacterial mode to initiate DNA replication. *FEMS Microbiol. Rev.* 26 355–374 12413665 10.1111/j.1574-6976.2002.tb00620.x

[B31] Mott M. L. Berger J. M. (2007). DNA replication initiation: mechanisms and regulation in bacteria. *Nat. Rev. Microbiol.* 5 343–354 10.1038/nrmicro1640 17435790

[B32] Myllykallio H. Lopez P. Lopez-Garcia P. Heilig R. Saurin W. Zivanovic Y. (2000). Bacterial mode of replication with eukaryotic-like machinery in a hyperthermophilic archaeon. *Science* 288 2212–2215 10.1126/science.288.5474.2212 10864870

[B33] Norais C. Hawkins M. Hartman A. L. Eisen J. A. Myllykallio H. Allers T. (2007). Genetic and physical mapping of DNA replication origins in *Haloferax volcanii*. *PLoS Genet.* 3:e77 10.1371/journal.pgen.0030077 PMC1868953 17511521

[B34] Pelve E. A. Lindas A. C. Knoppel A. Mira A. Bernander R. (2012). Four chromosome replication origins in the archaeon *Pyrobaculum calidifontis*. *Mol. Microbiol.* 85 986–995 10.1111/j.1365-2958.2012.08155.x 22812406

[B35] Pelve E. A. Martens-Habbena W. Stahl D. A. Bernander R. (2013). Mapping of active replication origins *in vivo* in thaum- and euryarchaeal replicons. *Mol. Microbiol.* 90 538–550 10.1111/mmi.12382 23991938

[B36] Rajewska M. Wegrzyn K. Konieczny I. (2012). AT-rich region and repeated sequences – the essential elements of replication origins of bacterial replicons. *FEMS Microbiol. Rev.* 36 408–434 10.1111/j.1574-6976.2011.00300.x 22092310

[B37] Robinson N. P. Bell S. D. (2005). Origins of DNA replication in the three domains of life. *FEBS J.* 272 3757–3766 10.1111/j.1742-4658.2005.04768.x 16045748

[B38] Robinson N. P. Bell S. D. (2007). Extrachromosomal element capture and the evolution of multiple replication origins in archaeal chromosomes. *Proc. Natl. Acad. Sci. U.S.A.* 104 5806–5811 10.1073/pnas.0700206104 17392430 PMC1851573

[B39] Robinson N. P. Blood K. A. McCallum S. A. Edwards P. A. Bell S. D. (2007). Sister chromatid junctions in the hyperthermophilic archaeon *Sulfolobus solfataricus*. *EMBO J.* 26 816–824 10.1038/sj.emboj.7601529 17255945 PMC1794387

[B40] Robinson N. P. Dionne I. Lundgren M. Marsh V. L. Bernander R. Bell S. D. (2004). Identification of two origins of replication in the single chromosome of the archaeon *Sulfolobus solfataricus*. *Cell* 116 25–38 10.1016/S0092-8674(03)01034-1 14718164

[B41] Samson R. Y. Xu Y. Gadelha C. Stone T. A. Faqiri J. N. Li D. (2013). Specificity and function of archaeal DNA replication initiator proteins. *Cell Rep.* 3 485–496 10.1016/j.celrep.2013.01.002 23375370 PMC3607249

[B42] Wu Z. Liu H. Liu J. Liu X. Xiang H. (2012). Diversity and evolution of multiple *orc/cdc6*-adjacent replication origins in haloarchaea. *BMC Genomics* 13:478 10.1186/1471-2164-13-478 PMC3528665 22978470

[B43] Wu Z. Liu J. Yang H. Liu H. Xiang H. (2014). Multiple replication origins with diverse control mechanisms in *Haloarcula hispanica*. *Nucleic Acids Res.* 42 2282–2294 10.1093/nar/gkt1214 24271389 PMC3936714

[B44] Zakrzewska-Czerwinska J. Jakimowicz D. Zawilak-Pawlik A. Messer W. (2007). Regulation of the initiation of chromosomal replication in bacteria. *FEMS Microbiol. Rev.* 31 378–387 10.1111/j.1574-6976.2007.00070.x 17459114

[B45] Zhang R. Zhang C. T. (2003). Multiple replication origins of the archaeon *Halobacterium* species NRC-1. *Biochem. Biophys. Res. Commun.* 302 728–734 10.1016/S0006-291X(03)00252-3 12646230

